# Clinical characteristics, predictors of immune reconstitution inflammatory syndrome and long-term prognosis in patients with Kaposi sarcoma

**DOI:** 10.1186/s12981-017-0156-9

**Published:** 2017-05-30

**Authors:** Patricia Volkow, Gabriela Cesarman-Maus, Pamela Garciadiego-Fossas, Enrique Rojas-Marin, Patricia Cornejo-Juárez

**Affiliations:** 1 Infectious Diseases Department, Instituto Nacional de Cancerología (INCan), Av. San Fernando No. 22, Col. Sección XVI, Deleg. Tlalpan, 14080 Mexico City, Mexico; 2 Hematology Department, Instituto Nacional de Cancerología (INCan), Mexico City, Mexico; 3 Radiology Department, Instituto Nacional de Cancerología (INCan), Mexico City, Mexico

**Keywords:** Kaposi sarcoma, Immune reconstitution inflammatory syndrome, Highly active antiretroviral therapy, Combined antiretroviral therapy, Thrombocytopenia, Pulmonary Kaposi sarcoma

## Abstract

**Objective:**

To investigate the predictive factors for the development of Kaposi sarcoma-related immune reconstitution inflammatory syndrome (KS-IRIS) and long-term prognosis in patients starting combined antiretroviral therapy (cART).

**Methods:**

We studied a retrospective-cohort of consecutive antiretroviral-naïve patients with KS initiating cART from January 2005 to December 2011 and followed through June 2013. KS-IRIS was defined as ≥2 of the following: abrupt increase in number of KS lesions, appearance or exacerbation of lung-opacities or lymphedema, concomitantly with an increase in CD4+ cell-count ≥50 cells/mm^3^ and a decrease of >1 log in viral-load once started cART. We compared individuals who met KS-IRIS criteria with those that did not and described the long-term follow-up.

**Results:**

We included 89 patients, 88 males; 35 (39%) developed KS-IRIS at a median of 10 weeks (IQR 4–16). KS-IRIS patients had more pulmonary-involvement (60% vs. 16.6% of patients; *p* < 0.0001), eight died attributed to pulmonary-KS. Thrombocytopenia <100,000/mm^3^ at follow-up occurred in 36% of KS-IRIS vs. 4% in non-KS-IRIS patients (*p* = 0.0002), 45% KS-IRIS patients with thrombocytopenia died, non without KS-IRIS. Chemotherapy (bleomicyn–vincristine) was more frequently prescribed in KS-IRIS patients (88.6% vs. 29.6%) with no differences in outcome; 80% of all patients achieve KS complete remission, 52% of them never received chemotherapy. No difference between groups in the long-term follow-up (mean 52.4 ± 27.4 months) was found, only one patient developed a secondary malignancy (1.12%).

**Conclusions:**

Lung-involvement was predictive of IRIS development. Thrombocytopenia in KS-IRIS patients at week 12 follow-up after cART initiation was associated with high mortality. Over a third of patients with KS achieve remission without chemotherapy. Individuals that survive the initial period of KS-IRIS adhere to cART had a good long-term prognosis.

**Electronic supplementary material:**

The online version of this article (doi:10.1186/s12981-017-0156-9) contains supplementary material, which is available to authorized users.

## Background

Kaposi sarcoma (KS) was first described in the nineteenth century. In 1981 it became one of the signs that marked the beginning of the AIDS epidemic. This enigmatic disease is now recognized as a cytokine-mediated angioproliferative disease [1, 2] caused by human herpes-virus 8 (HHV-8), also referred as Kaposi sarcoma-associated herpesvirus (KSHV) [3, 4] indeed HHV-8/KSHV fulfilled Koch principles for an infectious disease pathogen. KS course is unpredictable even in the combined antiretroviral therapy (cART) era, and it is still associated with significant mortality [5, 6].

The use of combined antiretroviral therapy (cART) has resulted in a dramatic reduction in opportunistic infections and mortality in patients with human immunodeficiency virus (HIV) infection [7, 8]. The major benefit of cART is the inhibition of viral replication that results in gradual restoration of the pathogen-specific immune response through partial recovery of the immune system [9, 10]. Initiation of cART, even in the presence of decreasing plasma HIV-1 RNA viral loads and rising CD4+ cell counts [9, 11], may lead to unexpected clinical deterioration which is known as immune reconstitution inflammatory syndrome (IRIS), [10, 12]. IRIS is attributed to “deregulation” of the reconstituting immune system and unmasking of pathogens or antigens that were previously present but clinically asymptomatic [9, 13–15]. IRIS has been reported to occur in 10‒30% of antiretroviral-naïve patients with a 5–30% mortality rate [5, 16]. In Africa, it has become a significant cause of morbidity and mortality in HIV-infected patients with access to cART [5, 17, 18]. IRIS has been described in association with different infectious processes [10, 19–21]. It has also been described within the context of KS and is characterized by abrupt exacerbation of KS in patients on cART (KS-IRIS) [13].

In the present study, we describe the prevalence and risk factors associated with the development of IRIS: clinical characteristics, type of therapy, outcome, markers of poor prognosis and long-term prognosis in patients with KS from a cohort of patients in Mexico initiating cART. We also estimated the incidence of secondary malignancies in long-term survivors.

## Methods

We performed a retrospective study of consecutive antiretroviral-naïve patients with KS who began cART between January 1, 2005 and December 31, 2011 at the National Cancer Institute of Mexico (INCan), a referral center for adult patients with AIDS-associated cancers. We included follow-up data up to June 30, 2013 provided the patients had >2 hospital-visits, and had not received systemic corticosteroids within the 2 months prior or concomitantly with the initiation of cART [22]. We obtained information on demographics, co-existence of other AIDS-defining events, cART regimen, CD4+ lymphocyte counts and viral load at HIV diagnosis and at 12 weeks follow-up.

We included the following clinical features related with KS: presence and extent of mucosal involvement, number of skin lesions (considered disseminated if there were > 20 lesions or affecting ≥ 2 regions of the body), the presence and extent of lymphedema, gastrointestinal involvement diagnosed by endoscopy and confirmed by biopsy, and lung involvement (lung opacities on chest X-ray and a negative gallium scan or typical lesions documented by bronchoscopy). We further classified patients accordingly to ACTG staging classification [23, 24], and compared each stage between the two groups and mortality.

### IRIS criteria

KS-IRIS was diagnosed in patients receiving cART with a reduction of at least 1 log10 of HIV-1 RNA and/or an increase of ≥50 cells/mm^3^ or ≥twofold rise in baseline CD4+ cell count associated with clinical abrupt worsening of previously existing KS (“paradoxical”) or the development of KS (“unmasked”) within the first 6 months after the initiation of cART [25].

KS exacerbation was defined with at least two of the following criteria: abrupt increase in the size or number of KS lesions, appearance or exacerbation of lymphedema, and the appearance or increase of otherwise unexplained, gallium-negative, lung opacities on chest X-rays after starting cART [5, 6, 16, 25, 26]. The agreement by two clinicians was necessary for the diagnosis of KS-IRIS. A radiologist blinded to the patients’ diagnoses evaluated the chest roentgenograms.

We compared patients who met KS-IRIS criteria with those that did not. We evaluated clinical features, imaging studies, the use of chemotherapy, laboratory tests and patient outcomes.

The evolution of KS was classified at the last hospital visit as a complete response (CR) when patients had no clinically evident cutaneous or mucosal lesions; partial response (PR) when patients had >50% decrease in the number and/or size of the original lesions, with no new lesions; stable disease when patients had <50% decrease in lesions and no new lesions; disease progression when patients had an increase in size and/or number of KS lesions; and relapse when a patient who had been classified previously as PR or CR had new lesions or increased in lesion’s size or number.

Survival and follow-up time was calculated from the date of HIV diagnosis to the date when the patient was last seen, or the date of the patient’s death. The cause of death was attributed to KS in the presence of respiratory insufficiency, hemoptysis, gastrointestinal bleeding, and/or airway obstruction and the remainder as HIV-related, non-HIV-related, or unknown. Date and cause of death was corroborated at the Mexican National Death Certificate Registry. No autopsies were performed.

The chemotherapy regimen used in these patients was based on bleomycin (10 U/m^2^) and vincristine (1.4 mg/m^2^) [27], and was prescribed by the treating physician (usually for patients with extensive skin lesions and lymphedema and/or gastrointestinal or pulmonary involvement).

### Statistical analysis

KS-IRIS and non-KS-IRIS groups were compared employing the Student *t* test for independent groups, the Fisher exact test, and the Mann–Whitney test or Χ^2^ test as appropriate. Time to development of KS-IRIS was analyzed by Kaplan–Meier survival curves, whereas predictors of mortality and KS-IRIS were tested using Cox proportional hazard model, the variables with a p ≤ 0.1 were included in this analysis. Independent risk factors were identified in multivariate analysis utilizing a forward step-wise procedure with the criterion, variable inclusion and *p* > 0.10 were removal from the model. *p* values ≤0.05 were considered statistically significant. Statistical analysis was performed utilizing STATA 14 software (STATA. Corp. College Station, TX, USA).

## Results

A total of 394 new patients with HIV were seen at the INCan during the study period. One hundred sixty one (41%) presented with KS; 109 (68%) were antiretroviral-naïve or had initiated cART within 6 months of their first clinic appointment. Two patients were excluded from the analysis because they received systemic corticosteroids and 18 were excluded since they had two or less hospital visits.

Eighty-nine patients (88 males and 1 female) were included: 35 patients (39.3%) met the criteria for KS-IRIS and were compared with the 54 (60.6%) individuals who did not develop KS-IRIS. In the KS-IRIS group, there were four patients classified as “unmasked” and 31 classified as “paradoxical”. The median time between the initiation of cART and KS-IRIS diagnosis was 10 weeks (IQR 4–16). For all male patients, the risk factor for HIV infection was men who have sex with men (MSM); the woman was infected through heterosexual intercourse.

Combined ART was based on Non-Nucleoside Reverse Transcriptase Inhibitors (NNRTI) in 45 patients (51%), protease inhibitors (PI) in 43 patients (48%), and there was one patient with three Nucleoside Reverse Transcriptase Inhibitors (NRTI), (*p* = 0.359). Clinical, ACTG staging and laboratory data are shown in Table 1. Mean age in the KS-IRIS group was 32 ± 7 years vs. 36 ± 8 years in the non-KS-IRIS group (*p* = 0.01). HIV was diagnosed at a median of 10 weeks (IQR 6–30 weeks) before the initiation of cART; median baseline CD4 count was 92 cells/mm^3^ (IQR 33–214 cells/mm^3^) and was 249 cells/mm^3^ (IQR 144–387 cells/mm^3^) at week 12 after starting cART. Median follow-up from the start of cART to the last hospital visit did not differ different between groups [20 months (IQR 6–46) in the KS-IRIS vs. 21.5 (IQR 11–33) in the non-KS-IRIS group, *p* = 0.9]. Seventy (81.5%) of the patients were in ACTG stage T1, with no difference among the stages between groups and in mortality (Table 1).

**Table 1:** Clinical and laboratory characteristics of patients with KS-IRIS- and non-KS-IRIS

Patient characteristics	IRIS-KS N = 35 (39%) (CI95% 29–50)	Non-IRIS-KS (N = 54) (60.6%) CI 95% 50–71)	All patients (N = 89)	p
Mean age ± 2 SD (years)	32 ± 7	36 ± 8	35 ± 8	0.01
Weeks from HIV diagnosis to cART (median, IQR)	10 (5‒33)	10 (6‒30)	10 (6‒30)	0.24
Median baseline CD4+ cells/mm^3^ (IQR)	82 (30–140)	110 (36‒230)	91 (34–204)	0.43
Median CD4+ cells/mm^3^ 12 weeks after cART initiation (IQR)	212 (89–357)	308 (159–391)	249 (144–387)	0.28
ACTG staging				
S0T0	0	7 (13%)	7 (7.8%)	0.18
S1T0	1 (4.4%)	11 (20%)	12 (13.5%)	
S0T1	19 (54%)	18 (33%)	37 (41.5%)	
S1T1	15 (42.8%)	18 (33%)	33 (37%)	
Deaths ACTG staging				
S0T0	0	0	0	0.3
S1T0	0	0	0	
S0T1	5 (12.8%)	1 (1.1%)	6 (0%)	
S1T1	4 (10.2%)	4 (7.4%)	8 (9%)	
Median HIV-RNA copies/mm^3^ at baseline. (IQR)	240,000 (106,000–612,000)	84,731 (31,300–250,000)	131,818 (57,200–361,124)	0.02
HIV-RNA at follow-up^a^: copies/mm^3^				
Number of patients	31	50	81	0.02
<100	25 (80.6)	48 (96)	73 (90.1)	
101‒1000	4 (12.91)	1 (2)	5 (6.2)	
1001‒50,000	1 (3.2)	1 (2)	2 (2.5)	
>50,000	1 (3.2)	0	1 (1.2)	
Antiretroviral therapy—No (%)				
NNRTI	20 (57)	25 (46)	45 (50.6)	0.36
PI	15 (42)	28 (51.8)	43 (48.3)	
3 NRTI	0	1 (1.85)	1 (1)	
Mucosal involvement—No (%)	27 (77)	35 (65)	62 (70)	0.25
Confluent/disseminated skin lesions—No (%)	25 (71)	25 (46)	50 (56)	0.01
Lymphedema—No (%)	15 (43)	14 (26)	29 (32)	0.09
Deaths—No (%)	5 (33%)	2 (14)	7 (24)	0.39
Gastrointestinal involvement—No (%)^b^	14 (61)	10 (37)	24 (48)	0.16
Deaths %	6 (43%)	4 (40%)	10 (42%)	0.41
Pulmonary involvement—No (%)	22 (63)	9 (17)	31 (35)	<0.001
Deaths^c^	8 (38%)	2 (22%)	10 (30%)	0.45
Platelets at first visit^d^				
>100,000/mm^3^	29 (85%)	46 (85)	75 (85%)	0.62
50‒100,000	2 (6%)	4 (7%)	6 (7%)	
<50,000	3 (8.9%)	4 (7%)	7 ((8%)	
Platelets at follow-up^e^				
>100,000/mm^3^	20 (64%)	48 (96%)	68 (84)	0.0003
50‒100,000	4 (13%)	2 (4)	6 (7)	
<50,000	7 (23%)	0	7 (8.6)	
Deaths first year	9 (26%)	5 (9.3%)	14 (15.7)	0.07
KS attributable deaths	8 (89%)	0	8 (57%)	

*IQR* interquartile range, *cARTc* combined antiretroviral therapy, *HIV* Human immunodeficiency syndrome, *NS* not significant, *SD* standard deviation, *NNRTI* Non-nucleoside reverse transcriptase inhibitor, *PI* protease inhibitor, *NRTI* nucleoside reverse transcriptase inhibitor

^a^Viral load at follow-up was measured between weeks 12 and 16 after initiation of cART; four patients died before follow-up of viral load determination

^b^Endoscopy was performed in 50 patients (21 Immune reconstitution inflammatory syndrome-Kaposi’s sarcoma [IRIS-KS] and in 29 with non-IRIS)

^c^Mortality for pulmonary involvement was evaluated in the first 48 weeks after cART initiation

^d^Complete blood counts (CBC) were not performed at the first visit in 2 patients

^e^In five patients, CBC was not performed at follow-up

There was a significant difference between groups at baseline regarding the extension and number of KS lesions, as well as the presence of pulmonary involvement (Table 1 **)** more common in the KS-IRIS group. Initial chest roentgenogram showed often disseminated reticular and nodular opacities, Gallium scans performed in 17 patients (14 with KS-IRIS and three with non-KS-IRIS) prompted by clinical symptoms and an abnormal chest X-ray were negative in nine and one respectively. Pleural effusion was also more frequent in patients in the KS-IRIS group compared to the non-KS-IRIS group (24% vs. 2%, *p* = 0.001).

The initial HIV viral load was significantly higher among patients with KS-IRIS (Table 1 include data and comparisons). By 12 weeks of cART, 73 patients (90%) had achieved a viral load <100 copies/ml (25/80.6% for KS-IRIS and 48/96% for non-KS-IRIS groups, respectively, p = 0.02). Thrombocytopenia (<100,000/mm^3^), which did not differ at baseline between groups, developed in 11 of 35 patients with KS-IRIS at the 12-week follow-up visit and almost half of them (45.5%) died, including five of the seven individuals with platelet counts <50,000/mm^3^. None of the patients in the non-KS-IRIS group had thrombocytopenia <50,000/mm^3^ at follow-up and only two (4%) had platelet counts between 50,000 and 100,000/mm^3^.

Chemotherapy was administered to 31 (88.5%) individuals in the KS-IRIS group and to 17 (31%) in the non-KS-IRIS group (*p* < 0.0001), with a mean number of chemotherapy cycles of 3.9 ± 2.3 and 3.8 ± 2 cycles respectively, (*p* = 0.8), (Table 2). Chemotherapy was well tolerated with no major adverse events recorded. Overall 80% of KS patients achieved CR, 23 patients (66%) of KS-IRIS group and 48 (89%) of non-KS-IRIS (p = 0.117); and PR in three (9%) and one (20%) patients respectively. One patient with PR abandoned cART after he had developed viral failure and relapsed 3 years later. In relation to complete remission there was no difference in outcome whether patient received chemotherapy versus no chemotherapy.

**Table 2:** Outcome of Kaposi sarcoma patients with IRIS and non-IRIS; treated with chemotherapy or without it, at time of last follow-up

	IRIS-KS (*N* = 35)			Non-IRIS-KS (*N* = 54)			Total (*N* = 89)		
	CR	PR	Death	CR	PR	Death	CR	PR	Death
Chemotherapy—*N* (%)	19 (54)	3 (9)^a^	9 (26)	15 (28)	0	2 (4)	34 (38)	3 (3)	11 (12)
No-chemotherapy *N* (%)	4 (11)	0	0	33 (61)	1 (2)	3 (5)	37 (42)	1 (1)	3 (3)
Total, *N* (%)	23 (65)	3 (9)	9 (26)	48 (89)	1 (2)	5 (9)	71 (80)	4 (4)	14 (16)

*IRIS-KS* immune reconstitution inflammatory syndrome-Kaposi’s sarcoma, *CR* complete response, *PR* partial response

^a^One patient abandoned treatment, had Kaposi’s sarcoma progression and died with AIDS and related comorbidities

**Fig. 1:**
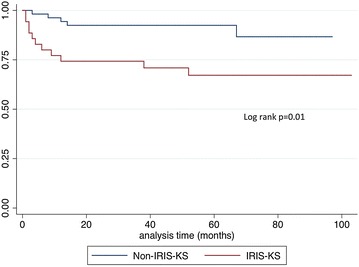
Kaplan-Meier survival curve. Overall survival is shown in months from combined active antiretroviral therapy (cART) initiation for patients with and without Kaposi’s sarcoma Immune Reconstruction Inflammatory Syndrome (KS-IRIS)

Sixteen patients (18%) died; eleven in the KS-IRIS group, eight of them were attributed to KS lung disease; with a median survival of 16 weeks (IQR 9‒50 weeks) after the initiation of cART. If patients survived the initial KS-IRIS phase and were adherent to cART, they had a good prognosis (p = 0.01). In the non- KS-IRIS group five patients died none related to KS, with a median survival time from cART initiation of 51 weeks (IQR 35–58) (Fig. 1).

Using Cox Proportional Hazard model the main predictor for the development of KS-IRIS was KS pulmonary involvement (HR 3.2, CI 95% 1.12–9.12, p = 0.02). Table 3 and only a platelet count bellow 100,000/mm^3^ at 12 weeks at follow-up after cART initiation was associated with death (HR 7.79, CI 95% 1.22–49.43, p < 0.02). Table 4.

**Table 3:** Cox regression model for IRIS development

Characteristics	**IRIS-KS (n** **=** **35)**	**Non-IRIS-KS (n** **=** **54)**	**Unadjusted HR (95% CI)**	**p value**	**Adjusted HR (95% CI)**	**p value**
IRIS						
HIV-RNA at follow-up^a^						
<100 copies/mm^3^	25 (81)	48 (96)	1.00			
>100 copies/mm^3^	6 (19)	2 (4)	5.76 (0.92–61)	0.04	2.26 (0.58–8.89)	0.239
Pulmonary involvement						
No	14 (40)	45 (83)	1.00			
Yes	21 (60)	9 (17)	7.5 (2.53–22.79)	<0.0001	3.20 (1.12-9.12)	0.02
Platelets at follow-up^b^						
>100,000/mm^3^	20 (65)	48 (96)	1.00			
<100,000/mm^3^	11 (35)	2 (4)	13.2 (2.46–128.61)	0.003	2.79 (0.92–8.43)	0.06

^a^Detectable viral load >1000 copies/mm^3^ at 12-week follow up. Viral load was documented in 81 patients

^b^Platelets <100,000/mm^3^ at 12-weeks follow-up. 81 patients had platelets count at follow-up

**Table 4:** Cox regression model for mortality

Characteristics	Death (n = 14)	Alive (n = 75)	Unadjusted HR (95% CI)	p value	Adjusted HR (95% CI)	p value
Mortality						
HIV-RNA at follow-up^a^						
<100 copies/mm^3^	6 (67)	67 (93)				
>100 copies/mm^3^	3 (33)	5 (7)	6.7 (0.81–44.41)	0.04	2.09 (0.33–13.23)	0.433
Platelets at follow-up^b^						
>100,000/mm^3^	6 (46)	62 (91)	1.00		1.00	
<100,000/mm^3^	7 (54)	6 (9)	10.16 (2.15–47.53)	0.001	7.79 (1.22–49.43)	0.02

^a^Detectable viral load >1000 copies/mm^3^ at 12-week follow up. Viral load was documented in 81 patients

^b^Platelets <100,000/mm^3^ at 12-weeks follow-up. 81 patients had platelets count at follow-up

The mean follow up of 81 patients surviving at least 26 weeks after cART initiation was 52.4 ± 27.4 months, for a total of 4.3 person/years for the whole group. Except for one case of Hodgkin’s lymphoma diagnosed 2 years after the diagnosis of KS, no other malignancies were diagnosed during follow-up. Incidence rate of secondary malignancy among patients with long-term follow up was 300 per 100,000 person-years.

Mean CD4 count at last visit was 400 ± 228 cells/mm^3^, 75 patients (93%) had an undetectable HIV viral load. Five patients had viral replication (median 100,000 copies/ml, IQR 1660–801,682); two of them had abandoned cART and for one patient data was unavailable.

There were no differences between KS-IRIS and non-KS-IRIS patients in the prevalence of KS as a sole AIDS defining event or coexistence of other AIDS defining illnesses. KS-related mortality was significantly higher among patients with KS-IRIS, but it was not different for patients with one or more defining AIDS events (Table 1).

## Discussion

KS remains the most frequent AIDS associated malignancy in HIV-infected individuals seen at the National Institute of Cancer in Mexico, representing 41% of all initial visits to the AIDS Clinic during 2005–2011. In this study we describe the prevalence of KS-IRIS in a group of patients starting cART, Pulmonary involvement was associated to the risk of developing KS-IRIS, and thrombocytopenia at 12 week’s follow-up after starting cART was associated to death. Over a third of patients with KS achieve remission without chemotherapy solely treated with cART.

The pathogenesis of KS is complex; it involves HHV-8/KSHV infection, cytokines and host-immune suppression [3, 28]. HHV-8 has oncogenic capacity, which mimics oncogenes that promote cell division, inhibit apoptosis, modulate inflammation and induce angiogenesis, but does not produce cell immortalization [29]; indeed spindle KS cells are not monoclonal. HHV-8 also induces viral and host cytokine production that promotes KS cell proliferation and differentiation (IL-6, oncostatin, alfa-TNF, PDGF, VEGF), and in HIV patients HIV-Tat protein also induces the expression of cytokines (N-kappa B activation, INF gamma, VEGF).

MSM with HIV or other active sexually transmitted diseases have the highest seropositive rates for HHV-8 [30]. At our hospital, only five women have been diagnosed with KS since 1990, and only one during the study period, consistent with the epidemiology reported in Mexico where KS is predominantly observed among MSM [31]. In our hospital we do not have HHV-8 serology.

In 1997, the first case of KS exacerbation in a patient who developed KS-induced airway obstruction 4 weeks after initiating cART was described [32]. To our knowledge, the rate of KS-IRIS found in our study is the highest reported to date (39% of patients) [33]. Our very high prevalence could be biased; since our institution is a tertiary-care center receiving patients with more extended KS disease [31]. The median time to development of KS-IRIS was 10 weeks, similar to the previously reported 6–14 week lapse after cART initiation [17, 19, 26, 33, 34]. IRIS associated to other pathogens (i.e., cytomegalovirus, MTB, MAC, and hepatitis B) has been reported to occur earlier within 2–5 weeks after cART initiation [33–35].

Higher HIV viral load was associated with the development of KS-IRIS by univariate analysis, which is a finding that had previously been reported though not consistently [33]. In two studies, an increase in CD4 count preceded KS progression in patients who had been severely immune-compromised before starting cART [4, 20, 35]. We also found an increase in CD4 counts at 12 weeks of follow-up, but this increase did not differ between the groups.

ACTG staging classification was not useful differentiating groups for IRIS-KS or mortality; we attribute this to the fact that most patients had extensive disease and low CD4.

cART has dramatically reduced opportunistic infections and mortality of HIV patients in established market-economy countries where early HIV diagnosis is promoted with extended access to HIV treatment [36, 37]. cART has also improved KS response and survival [38–42], it decreases HHV-8/KSHV viral load, and also produces complete regression of KS lesions with no additional therapy [8, 43]. However in countries such as Mexico with universal access to cART but where HIV diagnosis is delayed and patients arrive with severe immune suppression, [31] IRIS has increased the burden of mortality and morbidity of HIV-infected individuals [38].

Management of KS-IRIS has not been studied in controlled trials, but as opposed to some cases of *Mycobacterium* infection and cryptococcosis [9], corticosteroids are contraindicated in KS-IRIS, since it may further exacerbate KS lesions due to the synergy of cytokines with glucocorticoid receptors in KS spindle cells [22, 44, 45]. Herpes virus-specific immune reconstitution and the antiviral effect of combined cART plus chemotherapy has been recently documented with a trend toward better clinical outcome with cART plus chemotherapy compared with cART alone [46].

There are a wide variety of chemotherapy regimens published for KS [47–49]. In the present study chemotherapy was prescribed only for patients with more extended disease and outcome was not different between groups. It is important to note that 42% of patients achieving complete remission never received chemotherapy. The role of chemotherapy and its ultimate benefit in KS management remains a matter of debate and requires further research.

KS-IRIS is a time-limited phenomenon, but it can be a life-threatening one. Our experience shows that once the initial crisis is overcome, KS resolves in a high percentage of patients with limited or no chemotherapy and does not recur if patients maintain good adherence to cART.

The overall prevalence of pulmonary involvement in our series was higher (35%) compared to other studies (19–21%, respectively) [34, 50] likely reflecting sicker patients referred to our institution, and is one of the major risk for mortality described for KS-IRIS mortality [51, 52]. We found that pulmonary involvement was associated with the development of KS-IRIS, but not to death; only thrombocytopenia (<100,000 platelets/mm^3^) at 12 weeks of follow-up was significantly associated with patient demise.

The overall incidence of secondary malignancies observed in this population is lower to what has been reported in patients receiving pegylated liposomal doxorubicin (PLD), 1.2% vs. 9.2% at a long-term follow-up, median time 48 months (IQR 33–80) for patient surviving >26 weeks, vs. 50 months (IQ-17–76) after last PLD dose, respectively [53]. It is important to consider that PLD treated patients were slightly older (35 vs. 40 years), and the number of chemotherapy cycles administered was more than twice for PLD KS treated patients compared with our patients (9 vs. 4). Decrease in CD4 count while on PLD has been described [54], this fact needs further research to understand the role of PLD in the development of secondary neoplasia and its ultimate benefit in Kaposi’s therapy.

A limitation of the study that can contribute to the high prevalence of KS-IRIS we found, is that our institution is a referral tertiary care oncological center and only patients with more extensive disease are referred to our institution, while patients with few lesions and no organ involvement stay in primary care units for HIV patients. Other limitation is the analysis was retrospective and diagnosing a patient with KS-IRIS was based on published criteria [9, 13] with only two laboratory parameters and the rest mostly clinical. However, two experienced clinicians agreed on the diagnosis, and radiologic evaluation was blinded to clinical findings. Follow-up information was obtained from multiple sources such as the death certificates, pharmacy records, telephone calls and the National Program for Universal cART access, reducing uncertainty and biases.

Additional information on preventing and treating KS-IRIS is warranted including optimal timing of cART initiation for patients who present with extensive skin lesions and lung involvement, the impact of HIV and HHV-8/KSHV viral loads and CD4/CD8 counts as well as the production of inflammatory cytokines in diverse clinical scenarios as well as the role that other co-infections may have on the development of KS [55]. Anti-HHV-8/KSHV therapy should also be addressed, because both foscarnet and ganciclovir have proved activity in vitro [56] and epidemiological studies have shown that either antiviral given prophylactically for CMV infection diminishes the incidence of KS [57, 58].

It important to emphasize that abrupt clinical deterioration associated with IRIS does not represent cART failure; thus, a change of antiretroviral therapy is not warranted [26].

In summary KS patients with pulmonary involvement at diagnosis had higher risk of developing KS-IRIS. Thrombocytopenia at 12-week follow-up was significantly associated with mortality as determined by multivariate analysis. Over a third of patients with KS achieve remission without chemotherapy. Individuals that survive the initial period of KS-IRIS and adhere to cART had a good long-term prognosis. The information of the causes of death are available as Additional file 1: Table S1.

## Additional file

**Additional file 1:** **Table S1**. Time elapsed between cART initiation and death and cause of death.

## References

[1] Nakamura S, Salahuddin SZ, Biberfeld P, Ensoli B, Markham PD, Wong-Staal F (1988). Kaposi’s sarcoma cells: long-term culture with growth factor from retrovirus-infected CD4+ T cells. Science.

[2] Ensoli B, Nakamura S, Salahuddin SZ, Biberfeld P, Larsson L, Beaver B (1989). AIDS-Kaposi’s sarcoma-derived cells express cytokines with autocrine and paracrine growth effects. Science.

[3] Chang Y, Cesarman E, Pessin MS (1994). Identification of new herpesvirus like DNA sequences on AIDS-associated Kaposi’s sarcoma. Science.

[4] Chang Y, Parsonnet J (1999). KSHV, Kaposi’s sarcoma, and related lymphoproliferative disorders. Microbes and malignancy: infection as a cause of human cancers.

[5] Letang E, Lewis JJ, Bower M, Mosam A, Borok M, Campbell TB (2013). Immune reconstitution inflammatory syndrome associated with Kaposi sarcoma: higher incidence and mortality in Africa than in the UK. AIDS.

[6] Maskew M, Fox MP, van Cutsem G, Chu K, Macphail P, Boulle A, Egger M (2013). Treatment response and mortality among patients starting antiretroviral therapy with and without Kaposi sarcoma: a cohort study. PLoS ONE.

[7] Palella FJ, Delaney KM, Moorman AC, Loveless MO, Fuhrer J, Satten GA, Aschman DJ, Holmberg SD (1998). Declining morbidity and mortality among patients with advanced human immunodeficiency virus infection. HIV outpatient study investigators. N Engl J Med.

[8] Ledergerber B, Egger M, Erard V, Weber R, Hirschel B, Furrer H (1999). AIDS-related opportunistic illnesses occurring after initiation of potent antiretroviral therapy. JAMA.

[9] Murdoch DM, Venter W, van Rie A, Feldman W (2007). Immune reconstitution inflammatory syndrome (IRIS): review of common infectious manifestations and treatment options. (Abstract). AIDS Res Ther.

[10] Lawn SD, Bekker LG, Miller RF (2005). Immune reconstitution disease associated with mycobacterial infections in HIV-infected individuals receiving antiretrovirals. Lancet Infect.

[11] Shelburne SA, Visnegarwala F, Harcourt J, Graviss EA, Giordano TP, White C (2005). Incidence and risk factors for immune reconstitution inflammatory syndrome during highly active antiretroviral therapy. AIDS.

[12] Cooney EL (2002). Clinical indicators of immune restoration following highly active antiretroviral therapy. Clin Infect Dis.

[13] Bower M, Nelson M, Young AM, Thirlwell C, Newsom-Davis T, Mandalia S (2005). Immune reconstitution inflammatory syndrome associated with Kaposi’s sarcoma. J Clin Oncol.

[14] Shelburne SA, Hamill RJ, Rodríguez-Barradas MC, Greenberg SB, Atmar RL, Musher DW (2002). Immune reconstitution inflammatory syndrome: emergence of a unique syndrome during highly active antiretroviral therapy. Medicine.

[15] Lipman M, Breen R (2006). Immune reconstitution inflammatory syndrome in HIV. Curr Opin Infect Dis.

[16] Murdoch DM, Venter WD, Feldman C, Van Rie A (2008). Incidence and risk factors for the immune reconstitution inflammatory syndrome in HIV patients in South Africa: a prospective study. AIDS.

[17] Klotz SA, Aziz Mohammed A, Girmai Woldemichael M, Worku Mitku M, Handrich M (2009). Immune reconstitution inflammatory syndrome in a resource-poor setting. JIAPAC.

[18] Kambugu A, Meya DB, Rhein J, O’Brien M, Janoff EN, Ronald AR (2008). Outcomes of cryptococcal meningitis in Uganda before and after the availability of highly active antiretroviral therapy. Clin Infect Dis.

[19] Ratnam I, Chiu C, Kandala NB, Easterbrook PJ (2006). Incidence and risk factors for immune reconstitution inflammatory syndrome in an ethnically diverse HIV-1-infected cohort. Clin Infect Dis.

[20] Race EM, Adelson-Mitty J, Kriegel GR, Barlam TF, Reimann KA, Letvin NL (1998). Focal mycobacterial lymphadenitis following initiation of protease-inhibitor therapy in patients with advanced HIV-1 disease. Lancet.

[21] Park WB, Choe PG, Jo JH, Kim SH, Bang JH, Kim HB (2007). Tuberculosis manifested by immune reconstitution inflammatory syndrome during HAART. AIDS.

[22] Volkow PF, Cornejo P, Zinser JW, Ormsby CE, Reyes-Terán G (2008). Life-threatening exacerbation of Kaposi’s sarcoma after prednisone treatment for immune reconstitution inflammatory syndrome. AIDS.

[23] Chachoua A, Krigel R, Lafleur F, Ostreicher R, Speer M, Laubenstein L, Wernz J, Rubenstein P, Zang E, Friedman-Kien A (1989). Prognostic factors and staging classification of patients with epidemic Kaposi’s sarcoma. J Clin Oncol.

[24] Krown SE, Testa MA, Huang J (1997). AIDS-related Kaposi’s sarcoma: prospective validation of the AIDS Clinical Trials Group staging classification. AIDS Clinical Trials Group Oncology Committee. J Clin Oncol.

[25] French MA, Price P, Stone SF (2004). Immune restoration disease after antiretroviral therapy. AIDS.

[26] Nathan RV (2007). Suspected immune reconstitution inflammatory syndrome associated with the proliferation of Kaposi’s sarcoma during HAART. AIDS.

[27] Gompels MM, Hill A, Jenkins P, Peters B, Tomlinson D, Harris JR (1992). Kaposi’s sarcoma in HIV infection treated with vincristine and bleomycin. AIDS.

[28] Hengge UR, Ruzicka T, Tyrling SK, Stuschke M, Roggendorf M, Schwartz RA (2002). Update on Kaposi’s syndrome and other HHV8 associated diseases. Part 1: epidemiology, Environmental predispositions, clinical manifestations, and therapy. Lancet Infect Dis.

[29] Gantt S, Casper C (2011). Human herpesvirus 8-associated neoplasms: the roles of viral replication and antiviral treatment. Curr Opin Infect Dis.

[30] Hengge UR, Ruzicka T, Tyrling SK, Stuschke M, Roggendorf M, Schwartz RA (2001). Update on Kaposi’s syndrome and other HHV8 associated diseases. Part 1: epidemiology. Environmental predispositions, clinical manifestations, and therapy. Lancet Infect Dis.

[31] Crabtree-Ramírez B, Gómez-Palacio M, Rodríguez-Rivera V, Reyes-Terán G. Baseline characteristic of HIV-infected patients attending a National Institute of Health in Mexico City: evidence of late detection of HIV infection and the urgent need for efficient voluntary counseling and testing (VCT) programs. AIDS 2008. THPEO244 International AIDS Conference; August 2008. p. 390.

[32] Weir A, Wansbrough-Jones M (1997). Mucosal Kaposi’s sarcoma following protease inhibitor therapy in an HIV-infected patient. AIDS.

[33] Letang E, Almeida JM, Miró J, Ayala E, White I, Carrilho C (2010). Predictors of immune reconstitution inflammatory syndrome-associated with Kaposi sarcoma in Mozambique: a prospective study. J Acquir Immune Defic Syndr.

[34] Leidner RS, Aboulafia DM (2005). Recrudescent Kaposi’s sarcoma after initiation of HAART: a manifestation of immune reconstitution syndrome. AIDS Patient Care ST.

[35] Cheng VC, Yuen KY, Chan WM, Wong SS, Ma ES, Chan RM (2000). Immunorestitution disease involving the innate and adaptive response. Clin Infect Dis.

[36] Mocroft A, Kirk O, Clumeck N, Gargalianos-Kakolyris P, Trocha H, Chentsova N (2004). The changing pattern of Kaposi sarcoma in patients with HIV, 1994–2003: the EuroSIDA Study. Cancer.

[37] Eltom MA, Jemal A, Mbulaiteye SM, Devesa SS, Biggar RJ (2002). Trends in Kaposi’s sarcoma and non-Hodgkin’s lymphoma incidence in the United States from 1973 through 1998. J Natl Cancer Inst.

[38] Tam HK, Zhang ZF, Jacobson LP, Margolick JB, Chmiel JS, Rinaldo C (2002). Effect of highly active antiretroviral therapy on survival among HIV-infected men with Kaposi sarcoma or non-Hodgkin lymphoma. Int J Cancer.

[39] Paparizos VA, Kyriakis KP, Papastamopoulos V, Hadjivassiliou M, Stavrianeas WG (2002). Response of AIDS-associated Kaposi sarcoma to highly active antiretroviral therapy alone. J Acquir Immune Syndr.

[40] Nguyen HQ, Magaret AS, Kitahata MM, Van Rompaey SE, Wald A, Casper C (2008). Persistant Kaposi sarcoma in the era of highly active antiretroviral therapy: characterizing the predictors of clinical response. AIDS.

[41] Holkova B, Takeshita K, Cheng DM, Volm M, Wasserheit C, Dempoulos R (2001). Effect of highly active antiretroviral therapy on survival in patients with AIDS-associated pulmomary Kaposi’s sarcoma treated with chemotherapy. J Clin Oncol.

[42] Bower M, Wein J, Francis N, Newsom-Davis T, Powles S, Crook T (2009). The effect of HAART in 254 patients with AIDS-related Kaposi’s sarcoma. AIDS.

[43] Connick E, Kane MA, White IE, Ryder J, Campbell T (2004). Immune reconstitution inflammatory syndrome associated with Kaposi sarcoma during potent antiretroviral therapy. Clin Infect Dis.

[44] Guo WX, Antakly T (1995). AIDS-related Kaposi’s sarcoma: evidence for direct stimulatory effect of glucocorticoids on cell proliferation. Am J Pathol.

[45] Schulhafer EP, Grossman ME, Fagin G, Bell KE (1987). Steroid-induced Kaposi’s sarcoma in a patient with pre-AIDS. Am J Med.

[46] Bihl F, Mosam A, Henry LN, Chisholm JV, Dollard S, Gubi P (2007). Kaposi’s sarcoma-associated herpesvirus-specific immune reconstitution and antiviral effect of combined HAART/chemotherapy in HIV clade C-infected individuals with Kaposi’s sarcoma. AIDS.

[47] Newell M, Milliken S, Goldstein D, Lewis C, Boyle M, Dolan G (1998). A phase II study of liposomal doxorubicin in the treatment of HIV-related Kaposi’s sarcoma. Aust NZ J Med.

[48] Tulpule A, Groopman J, Saville MW, Harrington W, Friedman-Kien A, Espina BM (2002). Multicenter trial of low-dose paclitaxel in patients with advanced AIDS-related Kaposi sarcoma. Cancer.

[49] Cooley T, Henry D, Tonda M, Sun S, O’Connell M, Rackoff W (2007). A randomized, double-blind study of pegylated liposomal doxorubicin for the treatment of AIDS-related Kaposi’s sarcoma. Oncologist.

[50] Martin J, Laker M, Clutter D, Kambugu A, Janka D, Bennett J, et al. Kaposi’s sarcoma-associated immune reconstitution syndrome in Africa: initial findings from prospective evaluation. 16th Conference on Retrovirus and Opportunistic Infections 2009. Abstract #31.

[51] Crane HM, Deubner H, Huang JC, Swanson PE, Harrington RD (2005). Fatal Kaposi’s sarcoma-associated reconstitution following HAART initiation. Int J STD AIDS.

[52] Palmieri C, Dhillon T, Thirlwell C, Newsom-Davis T, Young AM, Nelson M (2006). Pulmonary Kaposi sarcoma in the era of highly active antiretroviral therapy. HIV Med.

[53] Martín-Carbonero L, Palacios R, Valencia E, Saballs P, Sirera G, Santos I (2008). Long-term prognosis of HIV-infected patients with Kaposi sarcoma treated with pegylated liposomal doxorubicin. Clin Infect Dis.

[54] Volkow P, Lizano M, Carrillo-García A, Pérez-Montiel D, Garciadiego P (2014). Triple secondary neoplasms: penis, lip and oral cavity in an AIDS patient treated with pegylated liposomal doxorubicin for cutaneous Kaposi’s sarcoma. AIDS.

[55] Feller L, Lemmer J (2008). Insights into pathogenic events of HIV-associated Kaposi sarcoma and immune reconstitution syndrome related Kaposi sarcoma. Infectious Agents Cancer.

[56] Neyts J, Clerq E (1997). Antiviral drug susceptibility of human herpesvirus. Antimicrob Agents Chemother.

[57] Mocroft A, Youle M, Gazzard B, Morcinek J, Halai R, Phillips A (1996). Anti-herpesvirus treatment and risk of Kaposi’s sarcoma in HIV infection. AIDS.

[58] Glesby MJ, Hoover DR, Weng S, Graham NM, Phair JP, Detels R (1996). Use of antiherpes drugs and the risk of Kaposi’s sarcoma: data from the Multicenter AIDS Cohort study. J Infect Dis.

