# Supplementary material for: Clinical characteristics, predictors of immune reconstitution inflammatory syndrome and long-term prognosis in patients with Kaposi sarcoma
Source: AIDS Res Ther. 2017 May 30;14:30. doi: 10.1186/s12981-017-0156-9 (PMC5450046; doi:10.1186/s12981-017-0156-9)
Supplement: Supplementary file 1 — Additional file 1: Table S1. Time elapsed between cART initiation and death and cause of death. [file 12981_2017_156_MOESM1_ESM.docx]

Table S1

| IRIS-KS (n=11) | Weeks from HAART initiation to death | Baseline viral load copies/mm^3^ | Follow-up viral load copies/mm^3^ | Follow-up CD4+  cells/mm^3^ | Follow-up platelets  cells/mm^3^. | Cause of death |
| --- | --- | --- | --- | --- | --- | --- |
|  | 26 | - | 423 | 36 | 216000 | Lung KS |
|  | 38 | - | 2690 | 33 | 107000 | Lung KS |
|  | 142 |  |  |  |  | Pneumococcus pneumonia, with KS relapse.  Patient abandoned HAART |
|  | 10 | 830,000 | - | 25 | 4000 | Lung KS |
|  | 5 | 14,100 | <40 | 19 | 21000 | Lung KS |
|  | 2 | - | <40 | 254 | 28000 | Lung KS |
|  | 50 | 89,424 | <40 | 461 | 36000 | Lung KS |
|  | 9 | 612,817 | <40 | 24 | 10000 | Lung KS |
|  | 11 | 209,817 |  | 78 | 39000 | Lung KS |
|  | 16 | 183,986 | <40 | 79 | 49000 | Kidney failure, nephrotic syndrome |
|  | 224 |  |  |  |  | Violence |
| Non-IRIS-KS (n=5) |  |  |  |  |  |  |
|  | 12 | 204,000 |  | 80 | 271000 | Toxic shock syndrome |
|  | 58 | 110,000 | 100,000 | 84 | 246000 | Lung abscess,  virologic failure |
|  | 251 | - | - | 230 | 281000 | Pneumonia  Patient abandoned HAART |
|  | 35 | 963,819 |  | 23 | 92000 | AIDS, MAC, disseminated candidiasis. |
|  | 51 | 153,127 |  | 83 | 321000 | AIDS, MAC |

*Sixteen patients (18%) died in the long term follow-up: 12 in the first year, mortality rate 13.5%; one in the second year, mortality rate 1.3%; one in the third, mortality rate 1.3%; and two in the fourth year after HAART initiation, mortality rate 2.6%. Patient with KS relapse had virologic failure and ultimately abandoned HAART. No cases of death due to secondary malignancy. Abbreviation MAC = mycobacterium avium complex
